# The effect of fascia gun on relaxation of exercise-induced muscle fatigue in athletes: a randomized controlled trial

**DOI:** 10.3389/fspor.2026.1831667

**Published:** 2026-06-03

**Authors:** Yutao He, Jianjun Yang, Xiangyu Jiang, Wanying Zhu, Chen Liang, Hengle Zhao, Yihui Ying

**Affiliations:** 1 Xizang Minzu University, Xianyang, China; 2 Qinghe Junior High School, Jiaxing, China

**Keywords:** athletes, exercise-induced muscle fatigue, fascia gun, muscle relaxation, percussion massage, percussive therapy, recovery, stretch

## Abstract

**Background:**

Inadequate recovery from exercise-induced muscle fatigue (EIMF) can negatively impact subsequent athletic performance. Although static stretching is widely used, its effectiveness remains controversial. The fascia gun, an emerging percussive massage tool, has been shown to alleviate pain, but evidence regarding its efficacy in promoting EIMF recovery compared to traditional stretching is limited. This study aims to compare the recovery effects of fascia gun percussive massage, static stretching, and passive recovery on exercise-induced muscle fatigue of the lower limbs in athletes.

**Methods:**

Thirty male athletes were recruited and randomly assigned to a fascia gun group, a static stretching group, or a blank control group (*n* = 10 per group). Lower limb fatigue was induced using squats at 60% of 1RM. Ratings of perceived exertion (RPE), thigh circumference, and muscle strength of the quadriceps, hamstrings, and gluteus maximus were measured before training, immediately post-fatigue, immediately post-intervention, and at 24 and 48 h post-intervention.

**Results:**

In the fascia gun group, RPE, thigh circumference, and the strength of all three measured muscles returned to pre-exercise levels at 24 h post-intervention. The stretching group required 48 h for recovery, while the blank control group had not fully recovered even after 48 h.

**Conclusion:**

Fascia gun percussive massage, by reducing perceived fatigue, alleviating muscle swelling, and accelerating muscle strength recovery, appears to promote recovery from exercise-induced muscle fatigue more effectively than static stretching in this study. It may serve as a practical rehabilitation tool for athletes.

## 1 Introduction

Exercise-induced muscle fatigue (EIMF) is an inevitable physiological phenomenon in sports training. If not addressed promptly and effectively, it can affect subsequent training quality and competitive performance for athletes.Article types (1).The most prominent symptom of EIMF is decreased muscle strength (2).This is a physiological protective mechanism that prevents the body from sustaining irreversible damage during exercise. EIMF arises from the complex interplay of multiple metabolic disturbances, including metabolic disorders, accumulation of metabolic byproducts, depletion of metabolic substrates, and alterations in the central nervous system’s recruitment strategy for motor units. Immediately upon the start of high-intensity exercise, phosphocreatine (PCr) is broken down to produce inorganic phosphate (Pi), which accumulates: this inhibits myosin ATPase activity and reduces cross-bridge formation (3). Between 10 s and 2 min, metabolic disturbances begin to appear, leading to depolarization of the resting membrane potential. This causes inactivation of voltage-sensitive sodium channels, resulting in a reduced action potential amplitude and decreased excitability. The reduced K^+^ and Na^+^ gradients induce a synergistic inhibitory effect, leading to decreased Ca^2+^ release from the sarcoplasmic reticulum and a decline in muscle strength. Increased Cl⁻ conductance further exacerbates K^+^-induced force inhibition (4). This stage primarily involves peripheral fatigue, manifested as cross-bridge dysfunction and insufficient energy supply. When exercise lasts for 2–30 min, the mechanisms underlying EIMF become more complex. Peripheral factors primarily manifest as accumulation of metabolic byproducts and substrate depletion. Large amounts of reactive oxygen species (ROS) and reactive nitrogen species (RNS) are generated, which inhibit Ca^2+^ release from the sarcoplasmic reticulum and reduce the sensitivity of myofibrils to Ca^2+^, thereby impairing excitation–contraction coupling. These factors also oxidatively modify contractile proteins such as myosin and troponin, leading to reduced muscle contractile force. Furthermore, they damage mitochondrial function, suppress aerobic metabolism, and accelerate the depletion of adenosine triphosphate (ATP) (5). As exercise proceeds, plasma levels of branched-chain amino acids (BCAAs) decline, reducing their competition with tryptophan for blood–brain barrier transport. This leads to increased tryptophan uptake into the brain, which in turn promotes the synthesis of 5-hydroxytryptamine (5-HT, also known as serotonin) (3). Concurrently, muscle contraction stimulates the production of cytokines such as IL-6. Together, these factors induce central fatigue, manifesting as feelings of weakness, reduced motivation to exercise, and decreased endurance. When exercise duration exceeds 30 min, central fatigue factors become dominant. Exercise-induced heat production exceeds heat dissipation, and the body's core temperature rises above 35.5°C. Brain temperature increases, cerebral blood flow decreases, and motor cortex excitability declines, further exacerbating central fatigue. Serotonin (5-HT) synthesis increases, leading to reduced motor output. Meanwhile, ammonia accumulates in the blood and crosses the blood–brain barrier, impairing brain mitochondrial function (6). Under the influence of central fatigue, the recruitment strategy of motor units is altered, resulting in a decreased level of muscle force output (7). The mechanisms underlying EIMF are complex; different mechanisms do not simply switch between each other but rather superimpose and interact. Central protective regulation exists from the very beginning of exercise, and the active decline in exercise intensity becomes more pronounced during the later stages of exercise.

The conventional protocol for active recovery involves performing exercise at an intensity lower than that of the preceding main workout immediately after the training session (8), However, for athletes already exhibiting symptoms of EIMF, this method cannot be used for effective relaxation. In American universities, 60.6% of collegiate athletes use stretching for post-exercise relaxation (9). Previous studies have clearly demonstrated that post-exercise stretching can improve symptoms of muscle contracture, thereby reducing muscle stiffness and increasing joint range of motion (9), reducing the risk of muscle strain (10), and can enhance athletes’ subsequent athletic performance and avoid the risk of injury in subsequent exercises (11).

The proposal of the fascia theory provides a new perspective for effective intervention after EIMF. This theory posits the existence of palpable, hypersensitive tight bands or nodules within the human body, known as myofascial trigger points (MTPs). When an MTP is pressed, it can produce significant local pain and may cause pain to radiate to other parts of the body.MTPs are classified as active or latent. Active MTPs spontaneously produce pain, elicit severe pain upon compression, and cause distinct referred pain. Biochemical changes (such as increased hydrogen ions and local ischemia) at latent MTPs, along with alterations in neural control, together create a local environment prone to rapid fatigue (12).

The fascia gun, as an emerging tool for muscle release, has been widely applied in the field of sports rehabilitation in recent years. Previous studies have shown that after using a fascia gun for percussive massage on MTPs, blood flow and skin temperature at the MTPs increase, myofascial relaxation occurs, soft tissue viscosity improves, joint range of motion increases, and the symptoms of exercise-induced muscle fatigue are effectively alleviated (13). Compared to traditional cooling and stretching, using a fascia gun to intervene in fatigued skeletal muscle has a lower requirement for body temperature. Percussive massage exhibits good intervention effects at different temperatures and can alleviate symptoms of muscle stiffness (14). However, according to previous systematic reviews, although percussive therapy can enhance muscle strength, power, and flexibility during subsequent exercise, and effectively alleviate skeletal muscle pain, the consistency of results across different RCT studies is low (15). Some studies have shown that percussive therapy and conventional stretching have similar effects. Therefore, previous intervention protocols need to be optimized to develop more effective intervention strategies for relieving exercise-induced muscle fatigue.

This study aims to compare the recovery effects of three modalities—fascia gun percussive massage, static stretching, and passive recovery—on exercise-induced muscle fatigue of the lower limbs in athletes through a randomized controlled trial. Research hypothesis: Compared with static stretching and passive recovery, fascia gun percussive massage can more rapidly reduce perceived fatigue, alleviate muscle swelling, and restore lower limb muscle strength.

## 2 Methods

### 2.1 Participants

*A priori* sample size estimation was performed using G*Power software (version 3.1.9.7; Universität Düsseldorf, Germany) for a mixed-design ANOVA (within-between interaction). The effect size was set at f = 0.25, corresponding to a moderate effect based on previous similar studies on percussive therapy and recovery (16). With an alpha level of 0.05, a power of 0.90, 3 groups, and 5 repeated measurements, the required total sample size was calculated to be approximately 27 participants. To account for an anticipated dropout rate of approximately 10%–15%, we recruited 30 male athletes, with 10 participants randomly allocated to each group.

A total of 30 male athletes were recruited from the School of Physical Education at Taizhou University and the School of Physical Education at Xizang Minzu University. Written informed consent was obtained from each adult participant prior to enrollment. All participants were aged ≥18 years and provided consent on their own behalf; no parental or guardian consent was required or obtained. Demographic data and sport-related information were collected through questionnaires and baseline measurements, including name, age, height, weight, athletic career duration, resting-state rating of perceived exertion (RPE), thigh circumference, and maximal isometric strength of the major lower limb muscles (quadriceps, hamstrings, and gluteus maximus).This study was approved by the Medical Ethics Committee of Xizang Minzu University (approval No. 2025-07-01, dated 1 July 2025). All procedures performed were in accordance with the ethical standards of the institutional research committee and with the 1964 Helsinki declaration and its later amendments.

The inclusion criteria for athletes participating in the experiment were as follows: (1) participation in competitions at the district/county level or above within the past year; (2) an athletic career duration of no less than 3 years; (3) current enrollment as a university physical education student. The exclusion criteria were: (1) presence of lower limb musculoskeletal injuries; (2) cessation of training for more than 3 months.

### 2.2 Study design

Participants were strictly restricted from engaging in any form of additional exercise during the experimental period and were required to refrain from any exercise for at least 5 days prior to the start of the experiment. Diet was not strictly controlled during the experimental period; participants followed their usual dietary habits. However, they were required to refrain from alcohol consumption and medication for 24 h before the experiment began.

Participants were randomly assigned to the blank control group, static stretching group, and fascia gun group in a 1:1:1 ratio using a computer-generated random number table. The randomization sequence was generated by a researcher not involved in the experiment implementation and was concealed in sequentially numbered, opaque envelopes. After participants completed baseline testing, a staff member opened the envelopes one at a time to inform participants of their group allocation. All outcome measurements were collected by researchers who were blinded to group allocation. Blinding was also maintained during the data entry and analysis phases.

### 2.3 Establishment of fatigue model

The fatigue induction protocol was established following the method of muscle fatigue modeling previously employed by McDonald (17) and N. S. Gregory (16). During the preliminary experiment, participants’ height, weight, and BMI were measured. Following a warm-up, each participant's one-repetition maximum (1RM) for the squat was estimated using the Brzycki formula based on the number of repetitions completed at a submaximal load. Brzycki formula: 1RM ≈ *ω*/(1.0278–0.0278r) (where *ω* represents the total barbell weight used during the squat, and r represents the maximum number of repetitions performed at that load).

The squat load was set at 60% of the participants’ one-repetition maximum (1RM). On the official experimental day, after a thorough warm-up, participants performed 7 sets of 10 repetitions of barbell squats, with a 2 min rest interval between sets. Each repetition was required to be completed within 7 s: 4 s for the downward eccentric phase (with the thighs lowering below the horizontal plane), followed by a 1 s pause at the bottom, then 1 s for the upward concentric phase, and a 1 s pause in the upright position. This sequence was repeated for 10 repetitions without interruption until failure.

### 2.4 Rehabilitation programs

Two trained staff members performed all stretching interventions. Both staff members underwent 4 hours of standardized training and passed a practical assessment prior to the formal experiment to ensure consistency in technique and minimize experimental error.

1. Stretch program

Participants in the stretching group were instructed to lie supine or prone on a yoga mat as required, and a trained staff member assisted them in performing specific stretching exercises for a total duration of 30 min. The targeted muscle groups included the anterior thigh, posterior thigh, and gluteal muscles. The protocol comprised 5 different stretching exercises, with each exercise performed for 3 sets per leg, lasting 1 min per set.

2. Percussive therapy program

Participants were instructed to lie supine or prone on a yoga mat as required for relaxation. A staff member used a Hyperice Hypervolt 2 Pro fascia gun equipped with a standard spherical head to relax the designated muscle groups. The targeted areas included the bilateral quadriceps, hamstrings, and gluteus maximus. Relaxation was performed for 5 min per area on each side, totaling 30 min. First, the fascia gun was set to speed 1 (approximately 20 Hz) and applied with slow gliding movements covering the entire muscle belly for extensive relaxation.

**Figure 1:**
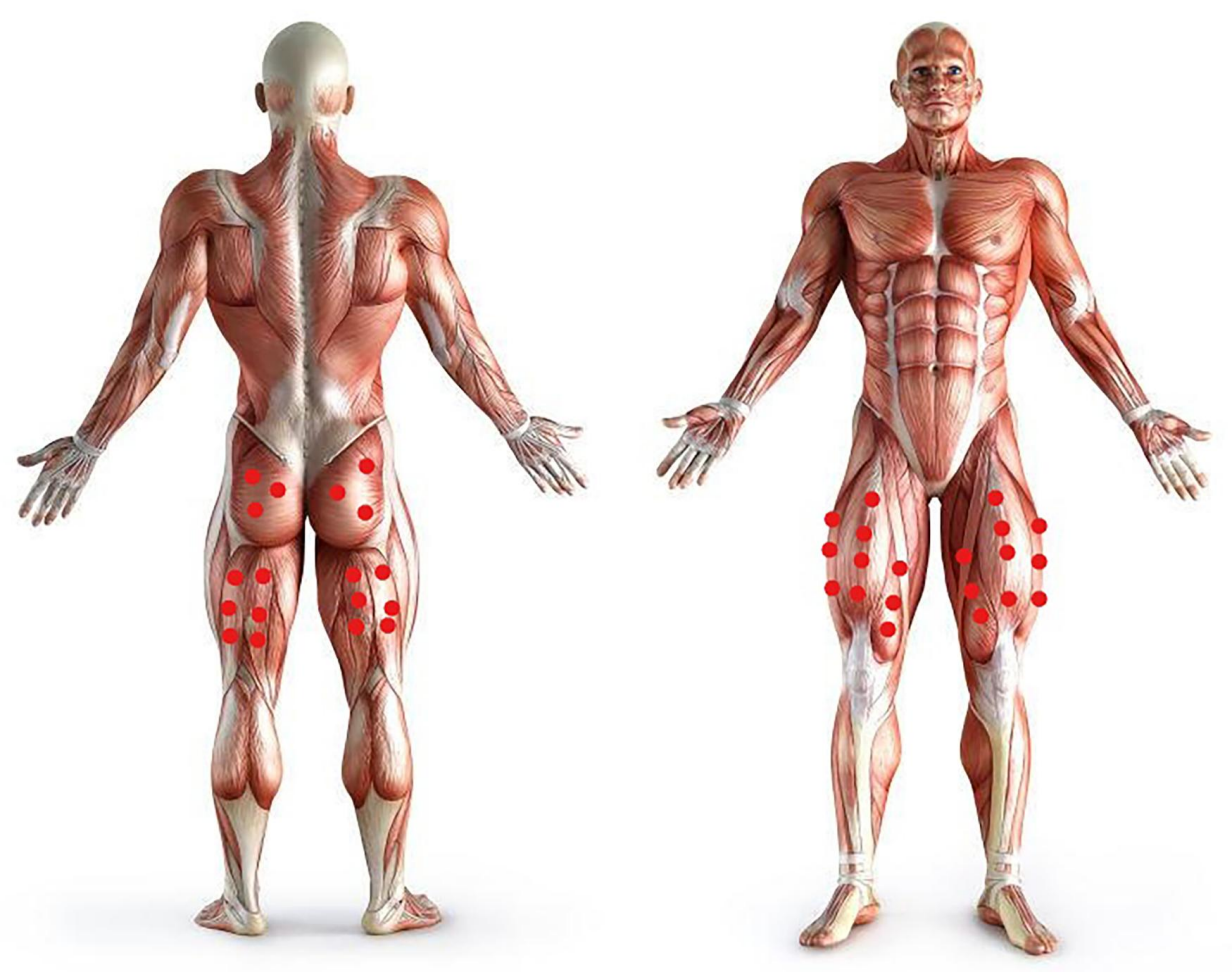
Trigger points of percussive therapy program.

The staff member first identified latent myofascial trigger points (MTPs) through palpation. Latent MTPs do not spontaneously produce pain; only upon compression do they elicit localized soreness or mild referred pain, and they are considered a core contributor to exercise-induced muscle fatigue (12). For trigger point treatment, the area was localized using a two-finger-width reference by the staff member. A fixed-point relaxation technique was applied, with continuous percussion on each point for 15–20 s, repeated 2–3 times to achieve the desired relaxation effect. The trigger points for fascia gun treatment are shown in Figure 1.

### 2.5 Outcome measurement

Outcome measurements were collected at five time points: before training (T0), immediately post-fatigue induction (T1), immediately post-intervention (T2), 24 h post-intervention (T3), and 48 h post-intervention (T4). The measurements included rating of perceived exertion (RPE), bilateral thigh circumference, and muscle strength of the quadriceps, hamstrings, and gluteus maximus.

The selected outcome measures were chosen to capture the multidimensional nature of EIMF recovery. RPE was selected as a validated psychophysiological measure of overall exertion and fatigue perception. Thigh circumference served as an indirect but practical indicator of exercise-induced muscle edema and swelling. Isometric muscle strength of the major lower limb muscle groups (quadriceps, hamstrings, gluteus maximus) was assessed as a direct measure of functional recovery, given that strength deficit is a primary symptom of EIMF and a critical determinant of subsequent athletic performance.

1. Rating of Perceived Exertion (RPE)

Rating of Perceived Exertion (RPE) was assessed using the RPE scale.

2. Thigh Circumference

**Figure 2:**
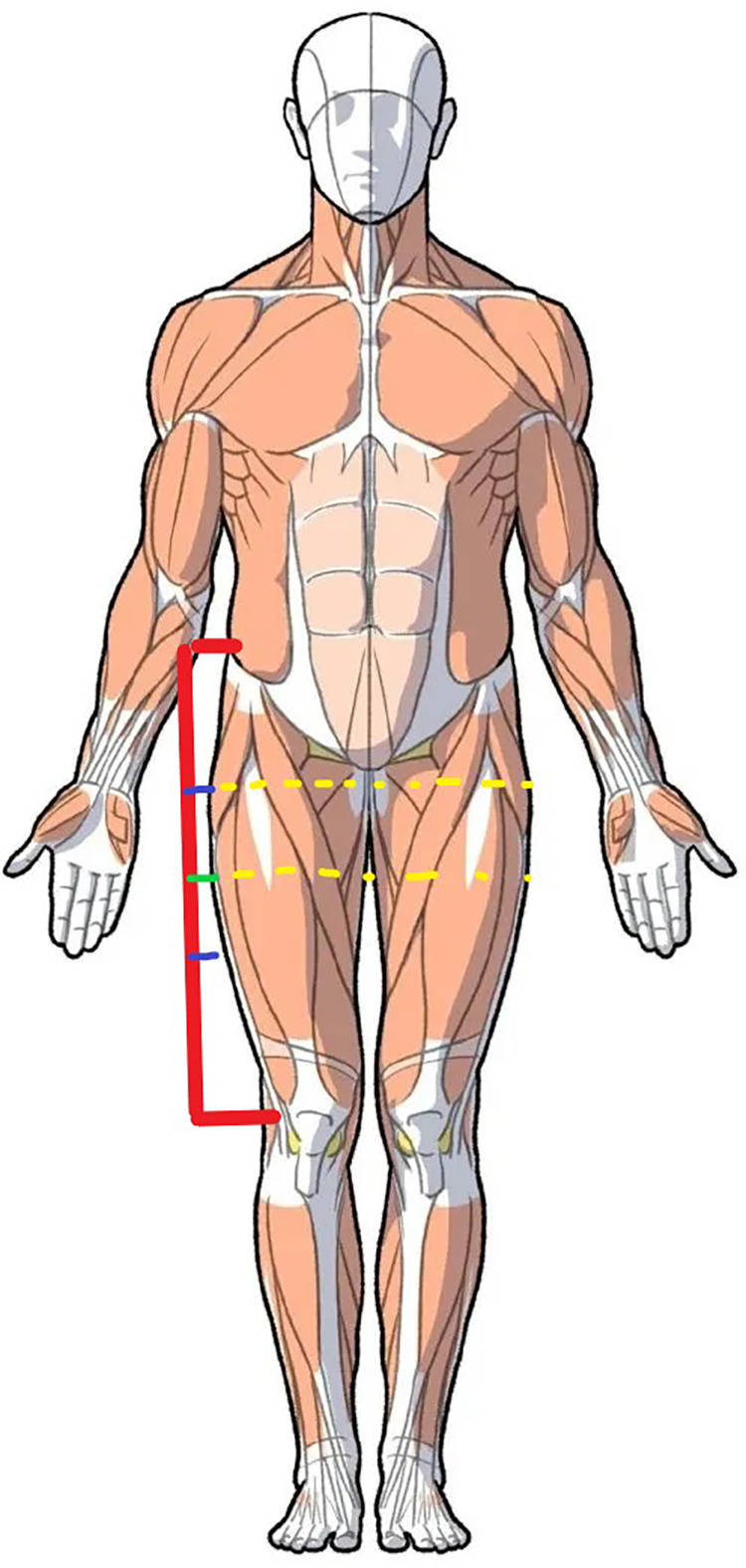
Thigh circumference measurement.

Participants lay supine on a yoga mat with their lower limbs relaxed. For the selection of a fixed measurement point for thigh circumference, the measurement range was between one-third and one-half of the distance from the anterior superior iliac spine (ASIS). During the first measurement, the staff identified the thickest point within this range, marked it with a marker, and used this marked point as a reference for all subsequent measurements. The same measuring tape was used consistently throughout the experiment. The maximum value was recorded, and each leg was measured twice to minimize error. The measurement procedure is illustrated in Figure 2.

1. Muscle Strength

A total of three muscles were tested: the quadriceps, hamstrings, and gluteus maximus. Muscle strength was assessed using a Hoggan MicroFET3 portable dynamometer (Hoggan Scientific, LLC). During testing, participants performed isometric contractions against the dynamometer for each target muscle. Two measurements were taken for each muscle, and the average value was calculated to minimize measurement error. During the measurements, straps were used, or an assistant firmly secured the participant's proximal body segments (e.g., pelvis, thigh) to allow only the target joint to move freely, thereby preventing compensatory movements and ensuring that the measured force was generated by the target muscle group.

### 2.6 Statistics

Statistical analyses were performed using SPSS version 26.0 (IBM Corp.). The Kolmogorov–Smirnov test was used to verify the normal distribution of all values. The study employed a 3 (group: control, stretch, fascia gun) ×5 (time: T0, T1, T2, T3, T4) mixed design, with group as a between-subjects factor and time as a within-subjects factor. A two-way repeated measures ANOVA (mixed ANOVA) was therefore conducted to examine the main effects of group and time, as well as the group×time interaction effect, for each outcome measure (RPE, thigh circumference, and muscle strength). One-way ANOVA was used for between-group comparisons at each time point when appropriate. Bonferroni correction was applied for *post-hoc* multiple comparisons to control for type I error. Statistical significance was set at *p* < 0.05. Data are presented as mean ± standard error (SE).

Prior to conducting the repeated measures ANOVA, the assumptions of sphericity, normality, and homogeneity of variances were tested. Sphericity was assessed using Mauchly's test of sphericity. If the sphericity assumption was violated (*p* < 0.05), the Greenhouse-Geisser correction was applied to adjust the degrees of freedom. Normality of the residuals was checked using the Kolmogorov–Smirnov test (*p* > 0.05 considered normal). Homogeneity of variances across groups at each time point was assessed using Levene's test. Effect sizes for the ANOVA results were reported using partial eta squared (*η*_p_^2^). According to Cohen's guidelines, *η*_p_^2^ values of 0.01, 0.06, and 0.14 were interpreted as small, medium, and large effects, respectively.

## 3 Results

### 3.1 Participant characteristics

According to the inclusion criteria and pre-intervention assessment standards, a total of 34 male athletes who met the study criteria were initially enrolled in the experimental intervention. Among them, 4 athletes withdrew from the trial due to personal reasons and were unable to complete the experimental procedures. A total of 30 athletes completed the full experimental protocol, including 10 participants in the blank control group, 10 in the static stretching group, and 10 in the percussive therapy group.

After one-way analysis of variance, there were no significant differences in age, height, weight, or BMI between the percussive therapy group and the control groups (*p* > 0.05). Participant characteristics are summarized in Table 1.

**Table 1:** Participant characteristics.

Characteristic	Control group (*n* = 10)	Stretch group (*n* = 10)	Fascia gun group (*n* = 10)	Total(*n* = 30)	F	*P*
Age(years)	20.80 ± 1.62	20.40 ± 1.07	21.30 ± 1.37	20.83 ± 1.37	1.10	0.35
Height(cm)	179.90 ± 5.95	182.7 ± 5.5	180.70 ± 5.36	175.53 ± 29.48	0.66	0.52
Weight(kg)	73.70 ± 4.55	73.60 ± 7.52	75.90 ± 7.64	74.40 ± 6.57	0.37	0.69
BMI(kg/m²)	22.82 ± 1.74	22.10 ± 2.57	23.21 ± 1.79	22.71 ± 2.05	0.74	0.49
Years of sport(years)	5.00 ± 0.82	5.60 ± 0.97	5.00 ± 1.15	5.20 ± 1.00	1.23	0.31

Resting-state levels were compared among the three groups, and no significant differences were observed in RPE, thigh circumference, or muscle strength of the quadriceps, hamstrings, and gluteus maximus at baseline (*p* > 0.05).

### 3.2 Subjective fatigue

A repeated measures ANOVA was conducted to analyze the subjective muscle fatigue levels of the 30 participants. Mauchly's test indicated a violation of sphericity (*p* < 0.05), therefore the Greenhouse-Geisser correction was applied. The results revealed a statistically significant interaction effect between group and time on RPE scores, F(4.52, 61.05) = 21.45, *p* < 0.01, *η*_p_^2^ = 0.614 (large effect). A significant main effect of group was also observed, F(2, 27) = 8.63, *p* < 0.01, *η*_p_^2^ = 0.390 (large effect). *post-hoc* comparisons with Bonferroni correction were subsequently performed.

Post-hoc comparisons with Bonferroni correction revealed that RPE scores in all three groups were significantly increased immediately after fatigue induction (T1) compared to baseline (T0) (*p* < 0.01). Immediately post-intervention (T2), RPE scores in all three groups remained significantly higher than at T0 (*p* < 0.05). At 24 h post-intervention (T3), RPE scores in the fascia gun group had returned to T0 levels (*p* > 0.05), whereas both the blank control group and the static stretching group showed significant differences compared to T2 (*p* < 0.01) but were still significantly different from T0 (*p* < 0.01). At 48 h post-intervention (T4), no significant differences in RPE scores were observed compared to T0 in any of the three groups (*p* > 0.05). These results indicate that fascia gun percussive massage enables subjective fatigue to return to baseline levels 24 h earlier than other interventions. Within-group differences are presented in Table 2.

**Table 2:** Rating of perceived exertion (RPE).

Groups	T0	T1	T2	T3	T4
Control (*n* = 10)	9.30 ± 1.49	15.20 ± 1.23^b^	14.70 ± 0.82^b^	14.30 ± 0.82^b^	9.00 ± 1.41^d^^,^^f^
Stretch (*n* = 10)	8.60 ± 1.58	15.40 ± 1.17^b^	14.90 ± 0.99^b^	14.30 ± 0.82^b^	9.00 ± 1.24^d^^,^^f^
Fascia Gun (*n* = 10)	8.90 ± 1.20	14.60 ± 0.70^b^	13.40 ± 0.97^b^^,^^d^	9.10 ± 1.45^d^^,^^f^	9.10 ± 1.45^d^^,^^f^

a Compare with T0 *p* < 0.05.

b Compare with T0 *p* < 0.01.

c Compare with T1 *p* < 0.05.

d Compare with T1 *p* < 0.01.

e Compare with T2 *p* < 0.05.

f Compare with T2 *p* < 0.01.

**Figure 3:**
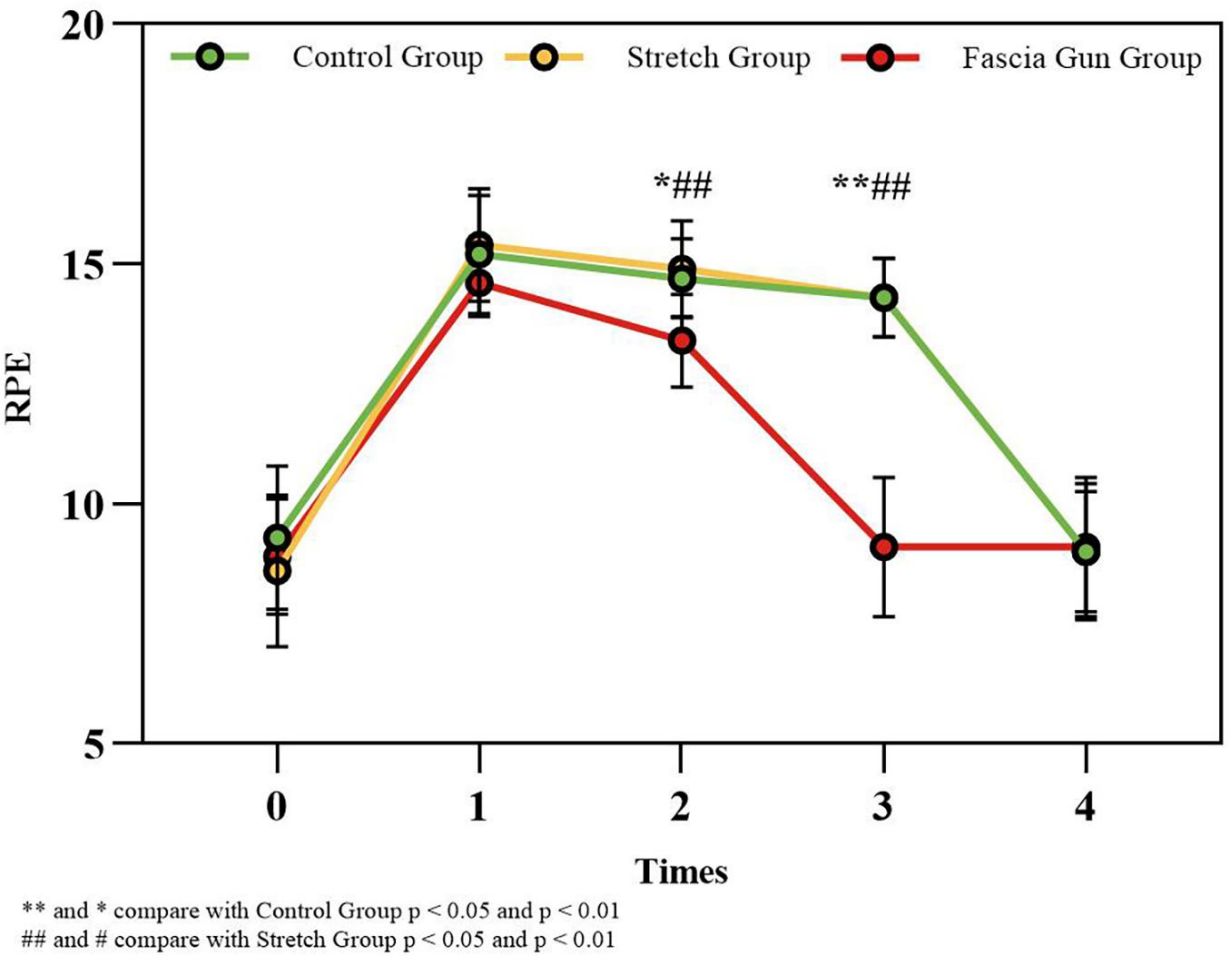
Rating of perceived exertion (RPE).

One-way ANOVA results indicated that at T2, RPE scores in the fascia gun group were significantly lower than those in the blank control group (*p* < 0.05) and the static stretching group (*p* < 0.01). At T3, RPE scores in the fascia gun group were highly significantly lower compared to the other two groups (*p* < 0.01). At T4, no significant differences in RPE scores were observed among the three groups (*p* > 0.05). Between-group differences are presented in Figure 3.

### 3.3 Muscle swelling

Thigh circumference was used as an indicator to assess delayed-onset muscle soreness and muscle swelling. Mauchly's test indicated a violation of sphericity (*p* < 0.05), therefore the Greenhouse-Geisser correction was applied. Repeated measures ANOVA revealed that the main effect of group was not statistically significant, F(2, 27) = 0.201, *p* = 0.819, *η*_p_^2^ = 0.015 (small effect). The main effect of side was also not statistically significant, indicating no significant difference in swelling between the two limbs, F(1, 27) = 0.135, *p* = 0.716, *η*_p_^2^ = 0.005 (large effect). However, the main effect of time was highly significant, indicating that time had a substantial impact on thigh circumference, F(1.59, 42.98) = 276.297, *p* < 0.01, *η*_p_^2^ = 0.911 (large effect).

Post-hoc comparisons revealed that at T1, thigh circumference in all three groups was significantly increased compared to T0 (*p* < 0.01). At T2, thigh circumference in all groups remained significantly higher than at T0 (*p* < 0.01). At T3, thigh circumference in the fascia gun group had returned to T0 levels (*p* > 0.05), whereas both the blank control group and the static stretching group showed significant differences compared to T2 (*p* < 0.01) but remained significantly different from T0 (*p* < 0.01). At T4, thigh circumference in the blank control group differed significantly from T2 (*p* < 0.01) but remained significantly different from T0 (*p* < 0.01); meanwhile, both the static stretching group and the fascia gun group had returned to T0 levels (*p* > 0.05). Within-group differences are presented in Table 3.

**Table 3:** Thigh circumference.

Groups	Sides	T0	T1	T2	T3	T4
Control (*n* = 10)	Left	53.34 ± 3.00	54.35 ± 3.03^b^	54.33 ± 2.98^b^	54.25 ± 2.97^b^	54.03 ± 2.96^b^
	Right	53.36 ± 3.03	54.30 ± 3.00^b^	54.34 ± 3.00^b^	54.17 ± 3.02^b^	54.04 ± 3.03^b^
Stretch (*n* = 10)	Left	53.73 ± 2.68	54.66 ± 3.05^b^	54.63 ± 3.05^b^	54.29 ± 3.02^b^^,^^d^^,^^f^	53.72 ± 2.70^d^^,^^f^
	Right	53.52 ± 3.32	54.50 ± 3.27^b^	54.49 ± 3.28^b^	54.24 ± 3.29^b^^,^^d^^,^^f^	53.53 ± 3.32^d^^,^^f^
Fascia Gun (*n* = 10)	Left	54.44 ± 2.78	55.54 ± 2.80^b^	55.17 ± 2.78^b^^,^^d^	54.46 ± 2.82^d^^,^^f^	54.47 ± 2.78^d^^,^^f^
	Right	54.46 ± 2.50	55.59 ± 2.61^b^	54.88 ± 2.58^b^^,^^d^	54.47 ± 2.40^d^^,^^f^	54.46 ± 2.40^d^^,^^f^

a Compare with T0 *p* < 0.05.

b Compare with T0 *p* < 0.01.

c Compare with T1 *p* < 0.05.

d Compare with T1 *p* < 0.01.

e Compare with T2 *p* < 0.05.

f Compare with T2 *p* < 0.01.

**Figure 4:**
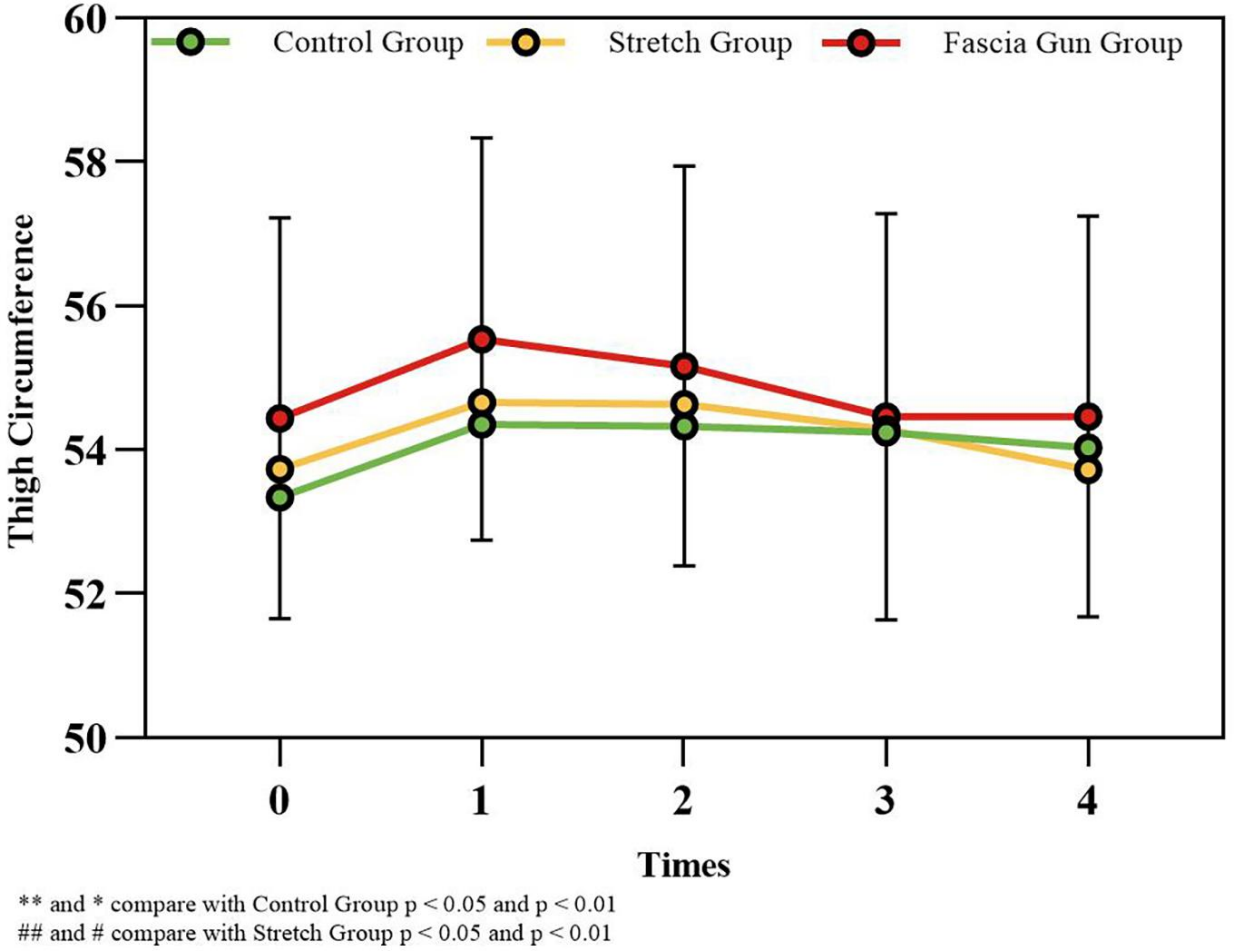
Thigh circumference.

One-way ANOVA results indicated that at all time points, there were no significant differences in thigh circumference among the three groups (*p* > 0.05). However, within-group analyses (Table 3) showed that the fascia gun group returned to baseline by T3, whereas the stretching group required until T4, and the control group had not recovered by T4. This suggests that although absolute swelling did not differ between groups, the rate of resolution was faster with percussive therapy. Between-group differences are presented in Figure 4.

### 3.4 Muscle strength

The recovery patterns of muscle strength were consistent across the three muscle groups (quadriceps, hamstrings, and gluteus maximus). For each muscle, repeated measures ANOVA revealed a significant main effect of time (*p* < 0.01), a significant main effect of group (*p* < 0.05), and a significant group × time interaction (*p* < 0.01), with large effect sizes (*η*_p_^2^ ranging from 0.567 to 0.965 for time effects, and 0.239 to 0.260 for group effects). The main effect of side was not significant for any muscle (*p* > 0.05), indicating symmetric recovery between limbs. Complete ANOVA results for each muscle are presented in Table 7.

**Figure 5:**
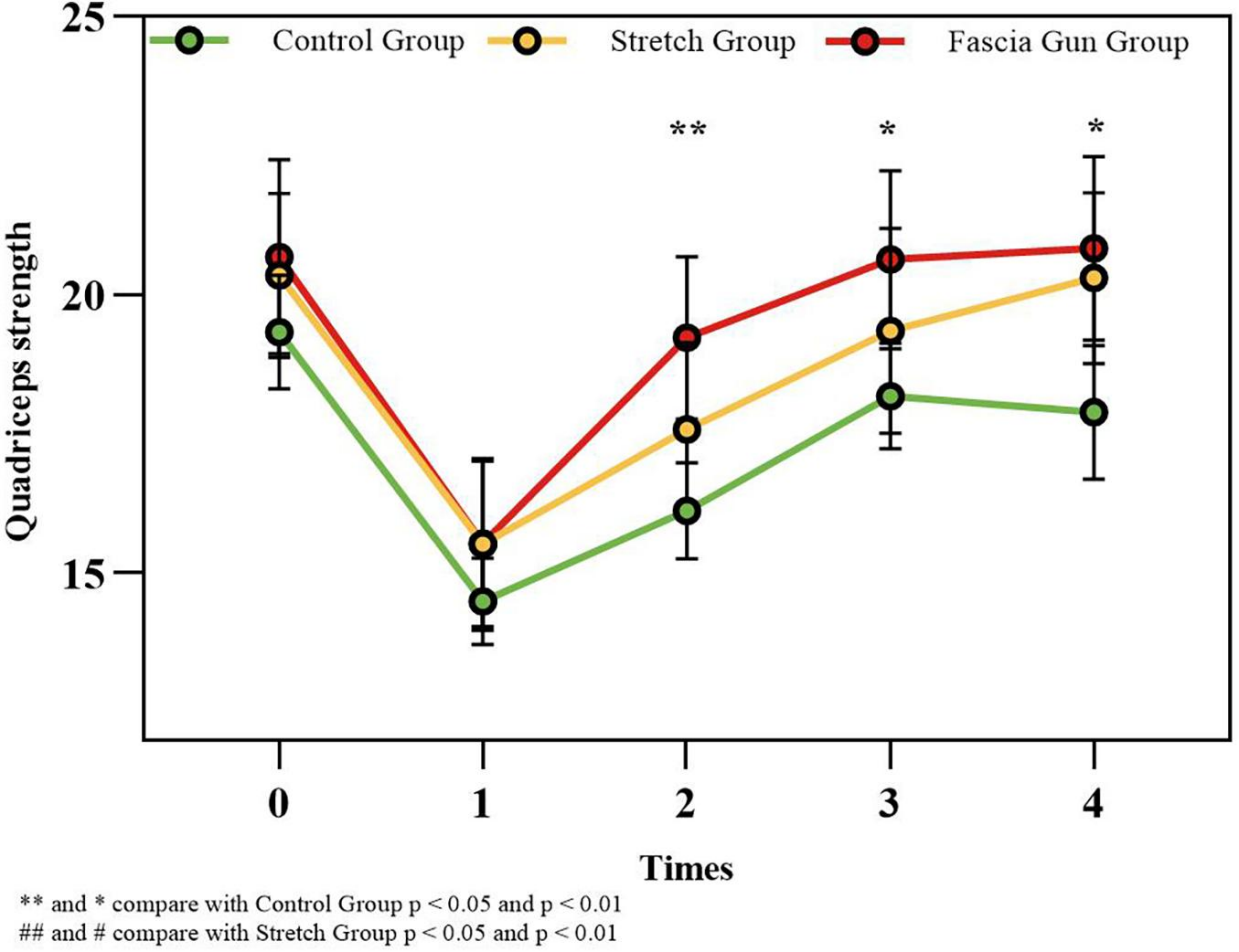
Quadriceps strength.

**Figure 6:**
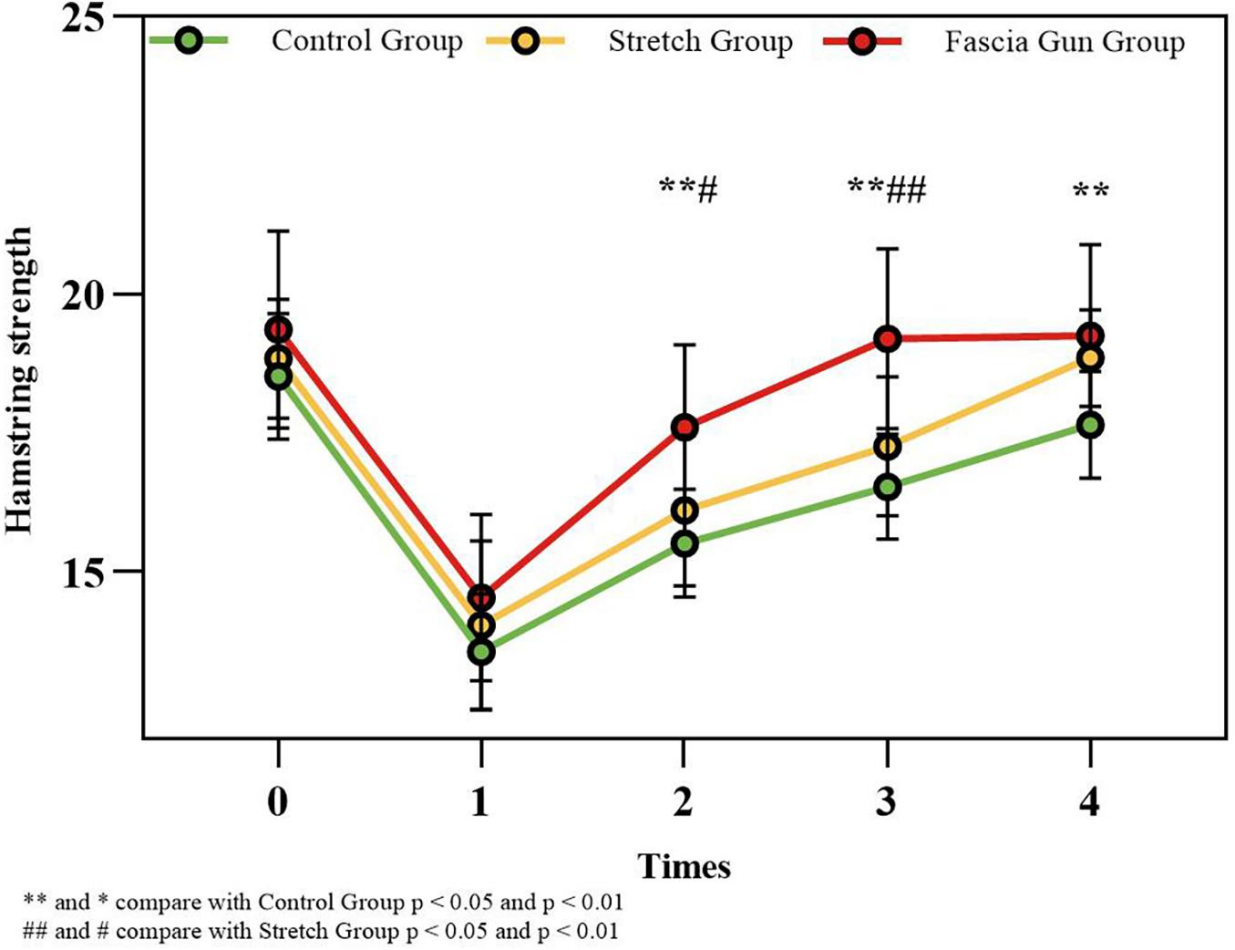
Hamstring strength.

**Figure 7:**
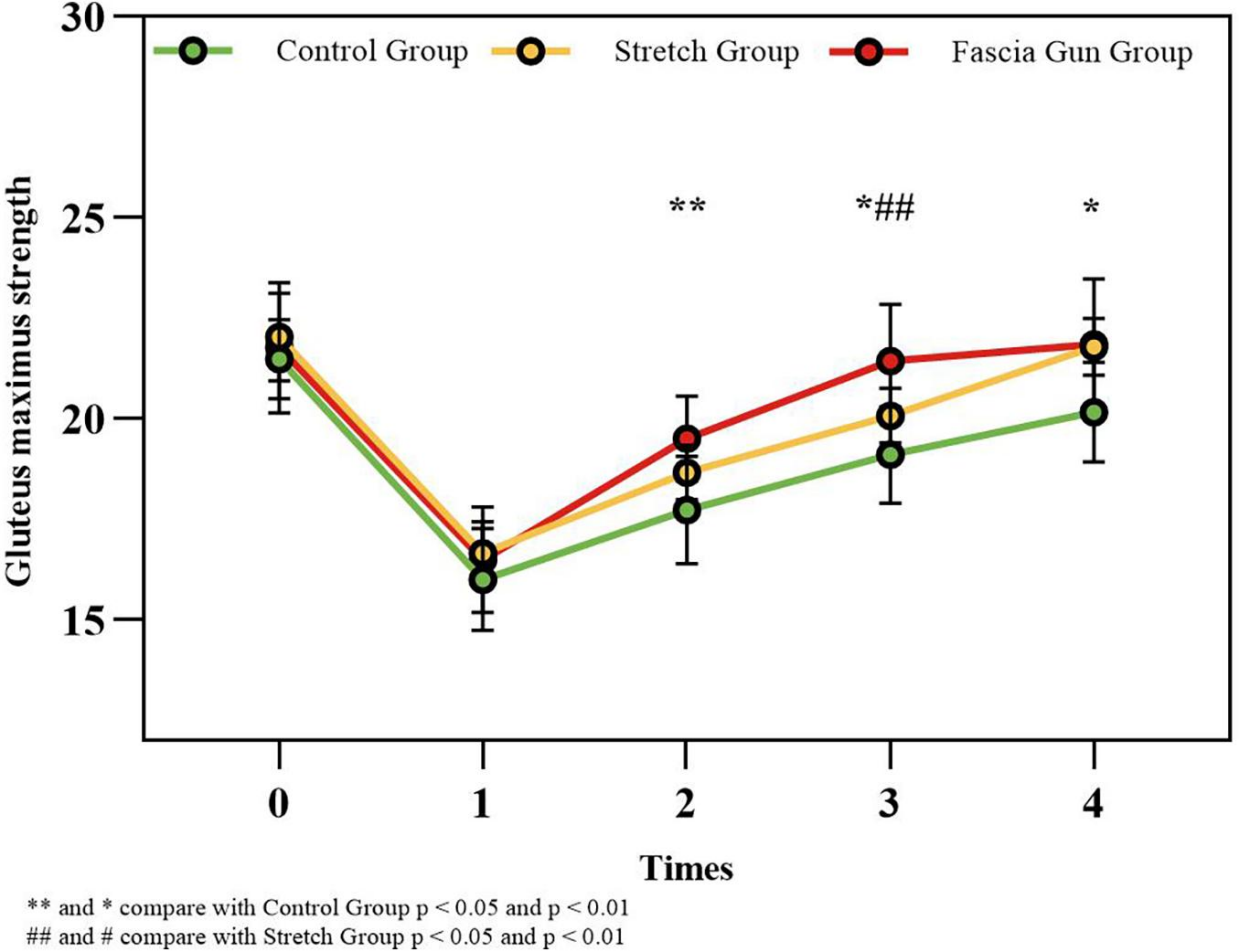
Gluteus maximus strength.

In the fascia gun group, muscle strength returned to baseline levels at 24 h post-intervention (T3) for all three muscles. In the static stretching group, strength returned to baseline at 48 h post-intervention (T4). In the blank control group, strength remained significantly below baseline even at 48 h. At T2 (immediately post-intervention) and T3 (24 h), the fascia gun group showed significantly greater strength than both the control and stretching groups (*p* < 0.05 or *p* < 0.01). By T4, no significant difference was observed between the fascia gun and stretching groups, but both were superior to the control group. Detailed within-group changes and *post-hoc* comparisons are presented in Tables 4–6, and between-group differences are shown in Figures 5–7.

**Table 4:** Quadriceps strength.

Groups	Sides	T0	T1	T2	T3	T4
Control (*n* = 10)	Left	19.33 ± 1.02	14.49 ± 0.78^b^	16.12 ± 0.86^b^^,^^d^	18.18 ± 0.95^b^^,^^d^^,^^f^	17.89 ± 1.20^b^^,^^d^^,^^f^
	Right	19.80 ± 1.37	14.85 ± 1.08^b^	16.56 ± 1.01^b^^,^^d^	18.52 ± 1.08^b^^,^^d^^,^^f^	18.53 ± 1.29^b^^,^^d^^,^^f^
Stretch (*n* = 10)	Left	20.15 ± 1.63	15.32 ± 1.66^b^	17.38 ± 1.72^b^^,^^d^	19.15 ± 2.27^b^^,^^f^	20.10 ± 1.71^f^
	Right	20.54 ± 1.30	15.75 ± 1.40^b^	17.36 ± 1.44^b^^,^^d^	18.83 ± 1.14^b^^,^^d^^,^^f^	20.53 ± 1.27^d^^,^^f^
Fascia Gun (*n* = 10)	Left	20.14 ± 1.66	14.98 ± 1.30^b^	18.68 ± 1.48^b^^,^^d^	20.08 ± 1.52^d^^,^^f^	20.28 ± 1.63^d^^,^^f^
	Right	20.60 ± 1.57	15.25 ± 1.40^b^	18.81 ± 1.56^b^^,^^d^	20.61 ± 1.47^d^^,^^f^	20.65 ± 1.50^d^^,^^f^

a Compare with T0 *p* < 0.05.

b Compare with T0 *p* < 0.01.

c Compare with T1 *p* < 0.05.

d Compare with T1 *p* < 0.01.

e Compare with T2 *p* < 0.05.

f Compare with T2 *p* < 0.01.

**Table 5:** Hamstring strength.

Groups	Sides	T0	T1	T2	T3	T4
Control (*n* = 10)	Left	18.52 ± 1.13	13.56 ± 1.04^b^	15.52 ± 0.97^b^^,^^d^	16.53 ± 0.94^b^^,^^d^^,^^f^	17.65 ± 0.96^b^^,^^d^^,^^f^
	Right	18.10 ± 1.31	13.29 ± 1.33^b^	15.32 ± 1.20^b^^,^^d^	16.45 ± 1.04^b^^,^^d^^,^^f^	17.22 ± 0.90^b^^,^^d^^,^^f^
Stretch (*n* = 10)	Left	18.84 ± 1.07	14.04 ± 1.52^b^	16.11 ± 1.36^b^^,^^d^	17.26 ± 1.25^b^^,^^d^^,^^f^	18.85 ± 0.87^d^^,^^f^
	Right	19.52 ± 1.89	14.16 ± 2.11^b^	16.37 ± 1.78^b^^,^^d^	17.87 ± 1.87^b^^,^^d^^,^^f^	19.54 ± 1.85^d^^,^^f^
Fascia Gun (*n* = 10)	Left	19.36 ± 1.77	14.54 ± 1.49^b^	17.60 ± 1.49^b^^,^^d^	19.20 ± 1.62^d^^,^^f^	19.26 ± 1.63^d^^,^^f^
	Right	18.91 ± 1.12	14.20 ± 1.51^b^	17.02 ± 1.21^b^^,^^d^	18.78 ± 1.16^d^^,^^f^	18.93 ± 1.17^d^^,^^f^

a Compare with T0 *p* < 0.05.

b Compare with T0 *p* < 0.01.

c Compare with T1 *p* < 0.05.

d Compare with T1 *p* < 0.01.

e Compare with T2 *p* < 0.05.

f Compare with T2 *p* < 0.01.

**Table 6:** Gluteus maximus strength.

Groups	Sides	T0	T1	T2	T3	T4
Control (*n* = 10)	Left	21.47 ± 0.98	16.00 ± 1.26^b^	17.72 ± 1.33^b^^,^^d^	19.10 ± 1.20^b^^,^^d^^,^^f^	20.16 ± 1.24^b^^,^^d^^,^^f^
	Right	21.30 ± 0.61	16.25 ± 1.23^b^	18.09 ± 1.17^b^^,^^d^	19.32 ± 1.01^b^^,^^d^^,^^f^	20.28 ± 0.77^b^^,^^d^^,^^f^
Stretch (*n* = 10)	Left	22.03 ± 1.09	16.65 ± 0.78^b^	18.65 ± 0.67^b^^,^^d^	20.07 ± 0.68^b^^,^^d^^,^^f^	21.78 ± 0.70^d^^,^^f^
	Right	22.34 ± 1.17	16.70 ± 1.60^b^	18.80 ± 1.70^b^^,^^d^	20.34 ± 1.50^b^^,^^d^^,^^f^	22.25 ± 1.30^d^^,^^f^
Fascia Gun (*n* = 10)	Left	21.76 ± 1.62	16.49 ± 1.31^b^	19.50 ± 1.06^b^^,^^d^	21.43 ± 1.40^d^^,^^f^	21.84 ± 1.62^d^^,^^f^
	Right	22.16 ± 1.53	16.93 ± 1.67^b^	19.77 ± 1.52^b^^,^^d^	21.85 ± 1.61^d^^,^^f^	22.01 ± 1.67^d^^,^^f^

a Compare with T0 *p* < 0.05.

b Compare with T0 *p* < 0.01.

c Compare with T1 *p* < 0.05.

d Compare with T1 *p* < 0.01.

e Compare with T2 *p* < 0.05.

f Compare with T2 *p* < 0.01.

**Table 7:** Summary of repeated measures ANOVA results for muscle strength.

Muscle	Effect	F (df)	*p*	*η*_p_^2^	Effect size
Quadriceps	Time	747.10 (2.55, 68.75)	<0.01	0.965	Large
	Group	4.23 (2, 27)	0.025	0.239	Large
	Group × Time	17.95 (5.09, 68.75)	<0.01	0.571	Large
Hamstring	Time	1,343.24 (1.89, 50.97)	<0.01	0.980	Large
	Group	4.75 (2, 27)	0.017	0.260	Large
	Group × Time	21.07 (3.78, 50.97)	<0.01	0.610	Large
Gluteus maximus	Time	1,078.08 (1.78, 48.10)	<0.01	0.976	Large
	Group	4.27 (2, 27)	0.025	0.240	Large
	Group × Time	17.66 (3.56, 48.10)	<0.01	0.567	Large

## 4 Discussion

### 4.1 Fascia gun percussion massage accelerates recovery from exercise-induced muscle fatigue

This study compared the recovery effects of fascia gun percussive massage, static stretching, and passive recovery on exercise-induced muscle fatigue of the lower limbs in athletes through a randomized controlled trial. The results demonstrated that fascia gun percussive massage enabled subjective fatigue, muscle swelling, and muscle strength to return to baseline levels 24 h earlier than the other interventions, with significantly superior effects compared to static stretching and passive recovery.

### 4.2 Acceleration of subjective fatigue recovery

Application of fascia gun percussive massage in athletes with EIMF facilitates a more rapid alleviation of the subjective sensation of fatigue. The increase in RPE is primarily attributed to muscle soreness and the inability to sustain muscle contraction efficiency. Manifestations of both subjective and objective fatigue emerged explosively between 13 and 24 min post-exercise (T2 period). Elevated levels of metabolic markers including ammonia (AMM), lactic acid (LAC), creatine kinase (CK), lactate dehydrogenase (LDH), cartilage oligomeric matrix protein (COMP), C-terminal telopeptide of type II collagen (CTX-II), calcium ions (Ca^2+^), and the pain indicator neuropeptide γ (NPγ) were observed in the serum of individuals with EIMF (18). NPγ induces muscle soreness in individuals with EIMF through the spinal dorsal horn, while the accumulation of anaerobic metabolites inhibits spinal motor neurons, leading to an alteration in motor unit recruitment strategies and a subsequent decrease in muscle fiber contraction efficiency (19).

The use of fascia gun percussive massage on fatigued muscles promotes improved blood circulation within the muscles through external mechanical force, thereby increasing blood oxygen saturation and facilitating the removal of metabolic waste and pain-inducing substances (20). Under the percussive massage of the fascia gun, large-diameter nerve fibers are stimulated and activated, thereby inhibiting pain signals and alleviating pain symptoms (21). After the clearance of metabolic waste and pain-inducing substances within the muscle, α-motor neurons are activated, thereby regulating the γ system to optimize neuromuscular function and promoting the recruitment of new motor units (20).

The use of fascia gun percussive massage can rapidly improve symptoms of joint mobility restriction caused by pain and muscle adhesion in athletes after a single treatment session (22). Further research revealed that after percussive massage therapy, ultrasound signals of the muscle fascia were significantly reduced, indicating a decrease in accumulated metabolic waste, accompanied by a significant improvement in patients’ pain symptoms (23). Findings from systematic reviews synthesizing evidence from multiple RCTs indicate that repeated application of percussive massage to muscle tissue can effectively alleviate symptoms of skeletal muscle pain (15). The rapid recovery of subjective fatigue observed in the fascia gun group can be collectively explained by these mechanisms.

### 4.3 Accelerated recovery from muscle swelling

The alleviation of muscle swelling serves as compelling evidence of metabolic waste removal by the bloodstream. Muscle edema is primarily caused by the accumulation of metabolic waste and inflammatory factors. Following exercise, the blood is unable to adequately transport the fluid produced from the breakdown of lactic acid within the muscle tissue, leading to compression of intramuscular blood vessels and subsequent insufficient blood supply (24), Simultaneously, the accumulation of inflammatory factors leads to local capillary dilation and increased permeability, resulting in substantial exudation of plasma proteins and fluid into the interstitial spaces and intracellular compartments of muscle tissue (25).

Although no significant differences were observed in the absolute values of thigh circumference among the three groups at any time point, the differences in within-group recovery patterns still hold clinical significance: in the fascia gun group, thigh circumference had returned to baseline levels at T3, whereas the other two groups had not yet recovered; by T4, the stretching group had recovered, while the blank control group remained unrecovered. This indicates that fascia gun percussive massage can accelerate the resolution of muscle swelling, although this acceleration was not reflected in the between-group differences in absolute values. Previous research has indicated that thigh circumference, as an indirect indicator of muscle swelling, may have measurement precision affected by factors such as subcutaneous fat and measurement site, making it difficult to detect small between-group differences (26). Secondly, anatomical differences among individuals may lead to greater variability in thigh circumference, potentially masking statistical differences between groups. Nevertheless, the faster within-group recovery rate observed in the fascia gun group still holds important clinical significance, suggesting that percussive massage may accelerate the clearance of metabolic waste and the resolution of inflammation. Previous studies have shown that after percussive massage with a fascia gun, local blood circulation in the muscles, particularly at MTPs, is improved, leading to positive changes in the metabolic state of local tissues (27).

Lower limb muscles naturally recover within 48–96 h based on the intensity and load of exercise (28). Previous studies have shown that after percussive massage with a fascia gun, local blood circulation in the muscles, particularly at MTPs, is improved, leading to positive changes in the metabolic state of local tissues (27). Metabolic waste is rapidly transported away from edematous muscle tissue by the blood, which can promptly and effectively alleviate the state of lower limb muscle edema. The results of this study are consistent with this, suggesting that the fascia gun accelerates the resolution of muscle swelling by promoting blood flow.

### 4.4 Accelerated recovery of muscle strength

Muscle strength is a crucial factor influencing athletic performance, and muscle weakness, as one of the primary symptoms of EIMF, significantly impairs subsequent athletic performance. Initially, inspiratory muscle fatigue activates a metaboreflex, leading to vasoconstriction in the limbs and reduced blood flow, thereby accelerating the onset of fatigue in the working muscles (29). During exercise, anaerobic metabolism within the working muscles leads to the gradual accumulation of lactic acid in the muscle tissue, resulting in a decrease in intracellular pH, disruption of ionic balance, inhibition of enzyme activity, and insufficient energy supply (19), The substantial accumulation of metabolic waste products, including lactic acid and inflammatory factors, contributes to impaired calcium release in muscle fatigue (30), At the molecular level, this is primarily manifested as impaired calcium ion (Ca^2+^) release from the sarcoplasmic reticulum (SR) in skeletal muscle fibers. Scientists have proposed three potential mechanisms: (1) High-frequency action potential (AP) stimulation may result in the accumulation of extracellular potassium ions (K^+^), thereby reducing the efficiency and amplitude of action potential activation; (2) In resting muscle fibers, most ATP is bound to magnesium ions (Mg^2+^). Fatigue reduces intracellular ATP content and increases free magnesium ion concentration, which impairs the opening efficiency of SR calcium channels; (3) When the sarcoplasm is exposed to phosphate, Ca^2+^). release from the SR in skeletal muscle fibers is persistently reduced, as inorganic phosphate enters the SR and precipitates with calcium ions, leading to a decrease in free Ca^2+^). and a subsequent reduction in the total amount of Ca^2+^). available for release (31). All three hypotheses point to the same outcome: a decrease in intracellular pH, disruption of ionic balance, inhibition of enzyme activity, and insufficient energy supply (19). Ultimately, this leads to alterations in neural motor unit recruitment strategies and a reduction in muscle contraction efficiency (7).

Fascia gun percussive massage improves blood circulation, accelerating the clearance of metabolic waste products (such as lactic acid and inorganic phosphate), thereby creating a favorable intracellular environment for normal calcium ion release. The impairment of calcium release gradually recovers as metabolic waste products are cleared; however, due to the nonlinear nature of the force-calcium relationship, the rate of muscle strength recovery varies at different stages (2). In the present study, muscle strength of the quadriceps, hamstrings, and gluteus maximus in the fascia gun group returned to baseline levels at 24 h post-intervention, which was significantly faster than that in the static stretching group (recovery at 48 h) and the blank control group (failure to recover at 48 h). This finding has important practical implications, indicating that the fascia gun can accelerate athletes’ ability to return to training and competition. Furthermore, the fascia gun not only demonstrated effective relaxation effects on the superficial quadriceps with its distinct muscle belly and origin-insertion points, but also produced comparable relaxation effects on the deep, fat-covered gluteus maximus. This suggests that the fascia gun can effectively relax muscles at varying depths and may serve as an effective modality for relieving exercise-induced muscle fatigue.

### 4.5 Mechanisms underlying the effects of percussive therapy

The fascia theory emphasizes that the human body functions as an integrated, interconnected network of connective tissue, in which no part exists in isolation from the system (12), Any deformation occurring in any part of this connective tissue network can have a negative impact on the overall structure. Over time, areas of fascial restriction can propagate throughout the body. A lack of flexibility and spontaneity in movement can lead to increased trauma, pain, and restriction, resulting in biomechanically inefficient and energy-consuming movement patterns and postures. When performing heavy-load exercises, repetitive single movements, or maintaining the same posture for extended periods, fascial adhesions may occur. Additionally, fascia can shorten, solidify, or thicken due to trauma following regular exercise, which can lead to injury, inflammation, and poor posture, ultimately affecting the body's physiological adaptability.

After locating the MTPs, the fascia gun is glided either along or across the direction of the muscle fibers, following their orientation. Initially, a treatment head with a larger contact area may be attached, and a higher speed setting (higher frequency) can be used to stimulate the superficial soft tissues. At this stage, pressure should be applied using only the weight of the fascia gun itself. When stimulating superficial soft tissues, applying excessive pressure beyond the weight of the fascia gun itself, combined with high-frequency percussion, may potentially damage deeper muscle tissues. However, the fascia gun has a built-in stroke (percussive amplitude) that limits the depth of impact within a certain range, thereby enabling targeted relaxation of superficial tissues. After the superficial tissues have been relaxed, the device can be adjusted to a lower speed setting (lower frequency) and fitted with a smaller contact area attachment. Moderate pressure may then be applied to penetrate and stimulate the deeper tissues. This phase primarily targets MTPs, as fascial adhesions often involve the muscle fibers enveloped by the fascia. For these deeper structures, reducing the contact area enhances the percussive effect. The lower frequency setting is used to prevent high-frequency impacts from causing secondary damage to the muscle fibers and fascia.

The mechanical stimulation of percussive massage can induce multiple physiological effects: (1) promoting interstitial fluid flow and accelerating metabolic waste clearance; (2) activating mechanoreceptors through pressure stimulation, thereby inhibiting pain signal transmission; (3) reducing soft tissue viscosity through periodic percussion; and (4) increasing local temperature through improved blood circulation, which enhances enzyme activity and accelerates energy metabolism recovery.

### 4.6 Limitation

This study has several limitations that should be acknowledged:

1. Generalizability to recreational populations

The participant population was limited to male athletes with a training background of at least three years and current engagement in competitive sports. This selection criterion, while ensuring homogeneity and enhancing internal validity, limits the generalizability of our findings to individuals who engage in recreational or leisure-time physical activity. Recreational populations may differ significantly from trained athletes in terms of baseline fitness level, neuromuscular adaptation, pain tolerance, and recovery capacity. Therefore, the recovery effects observed in this study may not directly translate to non-athlete populations. Future studies should include recreationally active individuals to determine whether the benefits of fascia gun percussive massage extend to broader populations with varying levels of physical activity.

2. Generalizability to female athletes:

The participant population was limited to male athletes, which restricts the generalizability of our findings to female athletes.Sex-related differences in muscle mass, hormonal profiles, and recovery kinetics may influence the response to percussive therapy. Therefore, our findings may not directly generalize to female athletes. Future studies should include female participants to examine whether the observed recovery benefits of fascia gun massage extend to both sexes. Future studies should include diverse populations across sex, age, and training status to validate the applicability of the intervention.

3. Sample size:

Although *a priori* sample size calculation was performed (*n* = 27), the final sample of 10 participants per group remains relatively small. This sample size may limit the ability to detect subtle but clinically meaningful differences between groups. Therefore, The primary purpose was to explore feasibility and estimate effect sizes, not to provide definitive conclusions. Future confirmatory trials with larger samples and prospective registration are needed.

4. Dietary and lifestyle control

Dietary intake and recovery activities (e.g., sleep quality, non-experimental physical activity) were not strictly standardized. Although participants were instructed to maintain their usual habits and avoid additional exercise, individual variations in diet and lifestyle may have influenced recovery kinetics. Future studies should incorporate dietary logs and actigraphy to better control for these confounding factors.

5. Outcome measures:

The outcome measures used in this study—RPE, thigh circumference, and isometric strength—were primarily subjective or indirect indicators of recovery. We did not collect blood samples to analyze biochemical markers of muscle damage (e.g., creatine kinase, lactate dehydrogenase) nor employ electromyography (EMG) to directly assess neuromuscular function. The lack of such objective physiological data limits our ability to elucidate the underlying mechanisms of percussive therapy. Future investigations should incorporate these complementary measures to provide a more comprehensive understanding of the recovery process.

Thigh circumference, used as an indirect indicator of muscle swelling, may be influenced by subcutaneous adipose tissue and measurement error. Future studies should employ advanced imaging techniques such as ultrasound or magnetic resonance imaging to more accurately assess morphological changes in muscle tissue.

6. Subjectivity in trigger point identification:

The identification of latent myofascial trigger points via palpation, while performed by trained staff, involves an inherent degree of subjectivity. Although we standardized training and maintained procedural checks, the consistency of pressure application and point localization may still vary, potentially introducing operator-dependent bias.

7. Fascia gun attachments:

The fascia gun attachments used in this study were of the same size for all participants. Future research should explore the effects of different attachment sizes on rehabilitation outcomes, as different head types may produce varying mechanical effects on superficial vs. deep tissues.

## 5 Conclusion

This pilot randomized controlled trial compared the effects of fascia gun percussive massage, static stretching, and passive recovery on exercise-induced muscle fatigue of the lower limbs in male athletes. The fascia gun group demonstrated faster recovery than both the static stretching and passive recovery groups, with RPE, thigh circumference, and muscle strength returning to baseline levels within 24 h post-intervention. In contrast, static stretching required 48 h for full recovery, and passive recovery did not achieve full recovery within the 48 h study period.

These preliminary findings suggest that a single session of fascia gun percussive massage may accelerate post-exercise recovery more effectively than static stretching, potentially offering a practical tool for athletes needing rapid return to training or competition. However, given the pilot nature and limitations, our results should be confirmed in larger, confirmatory trials that include both sexes and incorporate objective biochemical measures of muscle damage.

## Data Availability

The original contributions presented in the study are included in the article/Supplementary Material, further inquiries can be directed to the corresponding author.
